# Relationship between the Main Communities and Environments of an Urban River and Reservoir: Considering Integrated Structural and Functional Assessments of Ecosystems

**DOI:** 10.3390/ijerph15102302

**Published:** 2018-10-19

**Authors:** Dehao Tang, Xingjian Liu, Xutao Wang, Kedong Yin

**Affiliations:** 1 School of Marine Sciences, Sun Yat-Sen University, Guangzhou 510006, China; yinkd@mail.sysu.edu.cn; 2 Key Laboratory of Marine Resources and Coastal Engineering in Guangdong Province, Guangzhou 510006, China; 3 CAS Key Laboratory of Ocean and Marginal Sea Geology, South China Sea Institute of Oceanology, Guangzhou 510301, China; lxj@scsio.ac.cn; 4 Scientific Institute of Pearl River Water Resources Protection, Guangzhou 510611, China; awuhu@126.com

**Keywords:** community, water quality, sediment, eco-exergy, biodiversity

## Abstract

Rivers and reservoirs in urban areas have been associated with environmental quality problems because of the discharge of domestic waste into water bodies. However, the key effects and the extent to which environmental factors can influence the integrated structure and function of urban river ecosystems remain largely unknown. Here, a relationship model involving the species composition of the community and the various environmental factors related to the water and sediment was developed in the dry season (N) and the flood season (F) in both the urban Jiaomen River (JR) and the Baihuitian Reservoir (BR) of Guangzhou City. Canonical correspondence analysis was used to determine the spatiotemporal drivers of the phytoplankton, zooplankton and macrobenthic communities in the river and reservoir systems. The combination of the thermodynamic-oriented ecological indicators and the biodiversity measures reflected the integrated structure and function of the ecosystems. Overall, the plankton community composition was found to be largely determined by the nutrient concentrations and oxygen index, and the development of the macrobenthic communities was mainly restricted by organic matter and heavy metals. Based on the results of the integrated assessment, the structure and function of the JR ecosystem were superior to that of the BR, and the F period displayed healthier results than the N period. Moreover, the structural and functional statuses of the high eco-exergy grade communities (macrobenthic communities) in the ecosystem influenced the regional changes observed in the results of the integrated assessment. The significant seasonal variations in the plankton community affected the seasonal variations in the integrated assessment. The results of this study provide a scientific basis for the management and restoration of regional freshwater environments and ecosystems.

## 1. Introduction

Understanding how ecological processes are interconnected over multiple spatial scales, ranging from global patterns to local community structures, is extremely important for fundamental and applied ecological research [1]. Urban rivers and reservoirs are key components of urban landscapes and that their ecological structures are influenced by multiple anthropogenic and natural variables [2,3,4,5]. Rivers and lakes that are located in urban areas, including river branches, landscape parks, and reservoirs, are influenced by intensive anthropogenic activities, and these ecosystems are vital to the economy and society. Currently, treated domestic sewage totals 4.99 million tons/day [6] (in 2017), and a proportion of the domestic sewage produced in urban areas is still directly released into urban rivers; this issue has become a serious environmental problem and has attracted increasing concern from the Chinese government [7,8]. Guangzhou is a highly urbanised megacity in southern China that has a population of 13.5 million people [9] (in 2015). The temporal and spatial changes in urban rivers and reservoirs are also representative of their urban developmental histories [10,11]. 

Reliable ecosystem status assessments of urban environments are key, especially due to the mounting pressures from human activities. A plethora of evaluation tools currently exist to help with successful management; however, there is no consensus regarding which indices or models should be used by environmental managers to establish the quality of the structure and function of an ecosystem [12,13]. In terms of the long-term conservation and sustainable use of ecosystems, such an integrative assessment model requires knowledge of the structure and function of the natural systems, and how these processes respond to human activities is crucial to assessing the effectiveness of management decisions [14]. Urban rivers may experience high relative nutrient inputs due to atmospheric deposition, allochthonous inputs and/or sediment mobilisation from the surrounding catchment [3]. Water chemistry changes have implications for biotic communities, and even a minor change in the physico-chemical parameters can influence primary production [15]. Water quality assessments are generally fragmentary determinants of the freshwater ecosystem. Therefore, it is necessary to introduce sediment parameters, which can provide information about past pollution events, especially in certain types of water bodies [16].

To quantify the relationship between environmental variables and species, multivariate statistics are commonly used in the forecasting of ecological and environmental parameters. Researchers often use canonical correspondence analysis (CCA), linear correlation, spatial autocorrelation, and cross-correlation statistics to understand the spatial relationships among biotic and abiotic variables of interest [17,18]. Many environmental factors that influence urban river communities have been reported, including the pH, nutrients, organic matter (OM), oxygen, and potential risk of heavy metals in the water or sediment [11,19,20]. These studies have provided valuable information about the relationships among the internal components of urban river ecosystems and the surrounding habitats. However, little is known about the structure and function of the assemblages that constitute the communities of these ecosystems, and such structure and function information is an important aspect of a reliable ecosystem status assessment.

In addition to the influence of external environmental factors, the relationships between biodiversity and ecosystem functions, especially the functions involving ecosystem services [21], are complex and can take many forms; thus, such relationships have been widely debated [22]. Moreover, these relationships are important components of assessments of how functions are related to the ecosystem structure [23]. Biodiversity indexes are used to reflect the degrees of species diversity, uniformity and abundance in a biological community [24]. A biodiversity index can be used to determine the structural changes in a biological community or the stability of the ecosystem, and the associated calculation is relatively simple [25]. However, the relationship between biodiversity and ecosystem functionality remain a controversial tropic in academic fields [26,27]. Many researchers have established evaluation models based on the relationship between biodiversity and stability [28], and these studies have confirmed that ecosystems with higher biodiversity are more resistant and resilient to environmental changes. Therefore, a high biodiversity index can promote ecosystem functionality to a certain extent [29], and the index can be used to describe the structure of the system.

Ecosystem functionality has most often been approached using methods that measure biomass, primary production or nutrient cycling [30]. In the evaluation of the community structure of an ecosystem, not all species are identified and treated in a unified way. Therefore, the diversity of the species composition in the ecosystem and the degree of species evolution may not be adequately reflected [31]. Moreover, all biological systems are open thermodynamic systems, and complex ecosystems are characterised by the diversity of species, the heterogeneity within the space-time range, and the nonlinear correlations among components of the system [32]. In this sense, thermodynamic-oriented indicators, such as eco-exergy indexes that are seen as holistic indicators, suitably reflect the structure and function of a system using small-scale information, such as evolution grade and biomass information at the species level [33]. Descriptors that account for the transfer of energy, including the potential transfer or loss of genetic information, embrace ecosystem complexity rather than reduce it (as in thermodynamic approaches), and this approach is a novel way of addressing ecosystem functionality [34,35]. Thus, thermodynamic-oriented indicators can serve as useful and necessary supplements to integrated ecosystem assessments.

The stability, diversity and balance among the elements of a system are important indicators that can be used to measure the functional and structural states of the system, and they can provide the basis for exploring the energy flow and material circulation in the system [36]. Although the status of an ecosystem cannot be directly quantified, it can be indirectly assessed through the application of ecologically integrated analyses; additionally, a relatively healthy ecosystem generally displays a good balance between system efficiency and resilience [37]. From the perspective of constructed theory, the eco-exergy theory is based on a small-scale perspective of organism structure and information organisation; thus, it reflects the level of self-organisation of an ecosystem, as well as the corresponding system evolution [38]. However, biodiversity is based on the population level and species richness from a macroscopic perspective; thus, biodiversity reflects the degree of ecosystem stability [39]. To provide an overall and comprehensive assessment of the structural function of an ecosystem, the present study establishes an integrated assessment system [40] and highlights the temporal and spatial changes in the ecosystem.

The objectives of this paper are to (1) investigate the temporal and spatial distributions of environmental factors and species using a specific river-reservoir as an example, (2) perform integrated ecosystem assessments of the selected area by classifying the biodiversity and eco-exergy indexes of each community, and (3) develop a relationship model framework by analysing the correlations among environmental and ecological community modules.

## 2. Materials and Methods

### 2.1. Study Area and Sampling Sites

**Figure 1:**
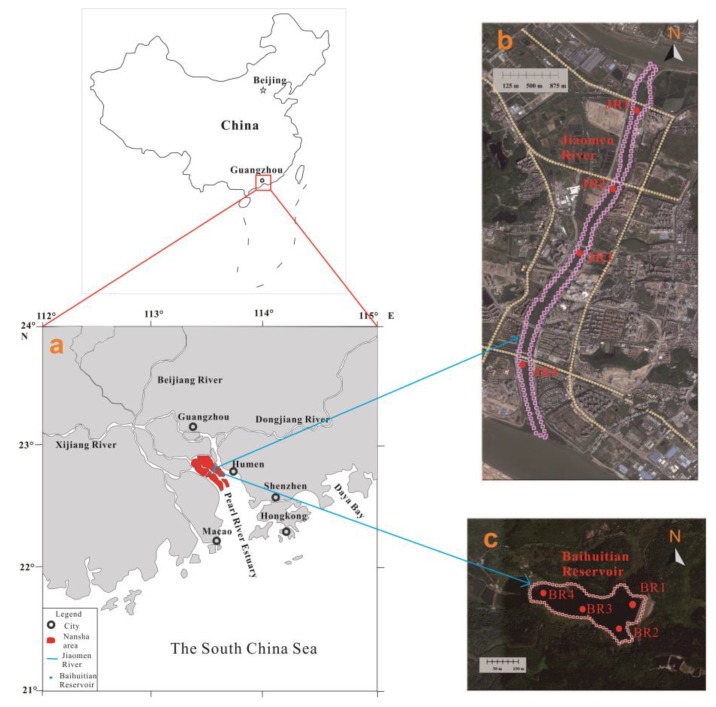
Map of the study area with locations of the sampling sites. (**a**) the Nansha Area; (**b**) the Jiaomen River; (**c**) the Baihuitian Reservoir.

We chose the Nansha Area (NA, Figure 1a) as the study area. The NA is a district in the southernmost region of Guangzhou City where the Pearl River flows into the South China Sea. The study area includes the confluence of three rivers: the Xijiang River, the Beijiang River and the Dongjiang River. These rivers correspond to three of the eight major outlets of the Pearl River: Humen, Jiaomen and Hongqili. This district is characteristic of the city and includes an extensive network of waterways; additionally, this district encompasses a water area of 188.15 km^2^, which accounts for 1/4 of the entire area. The Jiaomen River (JR) and Baihuitian Reservoir (BR) are two important water areas (Figure 1), and the ecological quality of these two water bodies has a direct impact on the safety of the drinking water in NA. The JR flows through the central part of NA and is a north-south river. Notably, the river includes the Xiaohuli Channel in the north and the Jiaomen Channel in the south, and the full length of the river is 6.43 km. The BR is located in Mount Huangshanlu Forest Park, and the reservoir has a water area of approximately 6.5 hectares and a capacity of 800,000 cubic metres. In the revision of the water source planning of Guangzhou City, the BR will be combined with the water resource allocation project of the Pearl River Delta as an emergency reserve water source for NA.

The samples were collected from the JR (Figure 1b) and BR (Figure 1c) in April (the non-flood period) and August (the flood period) of 2016, and each included four sampling sites (see Figure 1b,c). The sampling sites were selected based on the spatial distribution of human activities. Specifically, JR1–JR4 are located in areas with high population densities, which may result in a high contamination level; however, BR1–BR4 are located in the well-functioning environmental protection system of Mount Huangshanlu Forest Park, which theoretically reduces the probability of contamination. Replicates of both surface water and sediment samples were collected at each sampling site.

### 2.2. Environmental Sampling and Analysis

**Figure 2:**
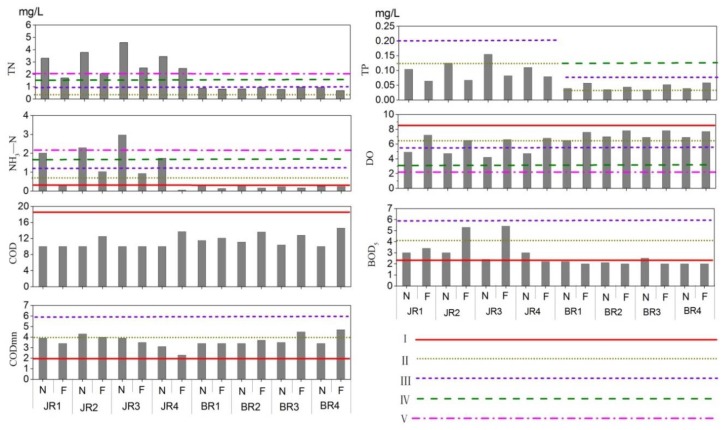
Monitoring values (mg/L) from the JR (Jiaomen River) and BR (Baihuitian Reservoir) and classification standards of water quality (WQ, *n* = non-flood season, F = flood season).

Water samples (500 mL, *n* = 3, taken from a depth of 0.5 m below the surface) were collected and stored in 500-mL polyethylene bottles that had been precleaned with deionized water and rinsed with the samples collected from the different sites. Water samples for nutrient (i.e., nitrate and phosphates) analysis were immediately placed on ice until further laboratory analyses, which occurred within 24 h of collection. Nutrient concentrations were determined using a multiparameter photometer. The pH, dissolved oxygen (DO), conductivity and water temperature were monitored in situ using a pH/DO meter. Environmental quality parameters, including total nitrogen (TN), total phosphorus (TP), the chemical oxygen demand (COD), the five-day biochemical oxygen demand (BOD_5_), the chemical oxygen demand (CODMn), and NH_3_-N, were measured according to the Environmental Quality Standard for Surface Water of PRC [41]. Five classes of surface water bodies (I–V, Figure 2, the threshold values and relevant explanation refer to the Supplementary Material, Table S1) were assessed based on their environmental functions and protection objectives, and these classes were identified by the Chinese government, based on the published “Environmental Quality Standards for Surface Water (EQSSW)” [41]. Specifically, Class Ⅰ is mainly assigned to source waters and national nature reserves; Class II is assigned to the centralised drinking water sources in first-order protection regions, rare aquatic habitats, the spawning grounds of fish and shrimp, and the feeding grounds of juvenile fish; Class III is assigned to drinking water sources in second-order protection regions, the wintering grounds of fish and shrimp, aquaculture areas, and swimming channels; Class IV is assigned to industrial use and recreational waters (which mainly refers to landscape water) that should not directly come into contact with the human body; and Class V is assigned to use for agricultural purposes only. According to the EQSSW, we used the water quality parameters, including TN, TP, DO, CODMn, COD, BOD_5_, and NH_3_-N, and the methods of the comprehensive water quality identification index to assess the water quality at all sampling sites in the JR and BR.

For sediment analysis, sediments (1.0 kg, *n* = 2, to a depth of ~5–10 cm) were collected at each site using a plastic hand shovel, and samples were transferred to polyethylene after the removal of overlying debris. The sediment samples were immediately placed into polyethylene bags and frozen at −20 °C before analysis. Each sample was divided into two portions, one for measuring nutrients (i.e., TP and TN) and OM, and the other for analysing heavy metals (Cd, Cr, Hg, Pb, As and Cu). The total content of OM in the sediments was estimated by the K_2_CrO_4_ external heating method using 0.3 g of dried sediment [42]. The contents of TN and TP in the sediments were determined using a KDY-9820 Kjeldahl analyser (Beijing, China) according to the micro-Kjeldahl method [43]. Freeze-dried samples were ground to pass through a size 200 mesh, and accurately weighted samples (~0.15 g for each sample) were digested with HCl-HNO_3_-HF-HClO_4_ in Teflon beakers. The concentrations of trace metals (Cr, As, Cu, Cd, Pb and Zn) were measured using an inductively coupled plasma-atomic emission spectrometer (ICP-MS, Agilent 7700×, Santa Clara, CA, USA). The Hg concentration in each sample was determined using a cold vapor atomic absorption spectrometer (CVAAS, Hydra-C). Quality control of the analysis was performed using a procedural blank, duplicates and a standard reference material GBW07309 (stream sediment) after every tenth sample. The elemental concentrations of the blanks were <1% of the mean analytical values for all metals. The determined concentrations of the metals in GBW07309 were within ±8% of the standard values.

### 2.3. Biological Sampling Collection

Biological community monitoring included the phytoplankton, zooplankton, and benthic communities.

The phytoplankton collection included qualitative and quantitative sampling. Qualitative samples were collected using plankton nets (made of size 25 sieve silk, pore size: 0.064 mm) that were towed in the water. Each net was towed several times until the water in the sample collector became unclear or coloured by the concentrated algae. Quantitative sampling was conducted using a 5000-mL water extractor; upper, middle and lower water samples were collected and then fully mixed, and a 1000-mL subsample was removed. The sampling amount was determined according to the actual conditions of the river sediment and the quantity of phytoplankton. Then, Lugol’s solution was added. The sample was allowed to settle for 48 h, concentrated to 100 mL, and inspected. Species identification was performed using a Carl Zeiss light microscope (Jena, THU, Germany), at 400 and 1000 magnification levels according to [44]. Phytoplankton were counted using perioptometry, and the quantity of phytoplankton in each litre of water was calculated as follows:

(1) $$N=\frac{C_{S}}{F_{S}\cdot F_{n}}\cdot \frac{V}{v}\cdot P_{n}\;$$

where N is the density of phytoplankton in 1 L of water (cells/L); C_s_ is the area of the settling cup (mm^2^); F_s_ is the area of the field of view under the microscope (mm^2^); F_n_ is the number of field views per slice; V is the volume of 1-L water samples after enrichment (mL); v is the volume of the settling cup (mL); and P_n_ is the number of counted cells. 

Zooplankton was collected using an organic glass hydrophore based on the actual conditions of the river and reservoir, and 5-L mixed-water samples was collected at intervals of 1 m below the water surface; thus, the average sample size was between 20 and 50 L. Water samples were filtered in situ using plankton nets (made of size 25 sieve silk, pore size: 0.064 mm), and samples were loaded into 200-mL transparent bottles and preserved in a 1% formaldehyde solution. The zooplankton abundance in each litre of water was calculated as follows:

(2) $$A=\frac{V_{c}}{V_{s}\cdot V_{m}}\cdot D$$

where A is the abundance of zooplankton in 1 L of water (ind./L); V_c_ is the volume of water samples after enrichment (mL); V_s_ is the sampling volume (L); V_m_ is the microscopic volume (mL); and D is the number of individuals counted.

There are three main types of benthos in the study area: insects, oligochaetes and molluscs. Qualitative samples are used to determine the species of organisms, while quantitative samples determine the number and biomass of organisms. Quantitative samples were collected using a sediment collector, and 2 samples were collected at each site. The qualitative samples of molluscs were collected using a D-shaped kick-net, and the qualitative samples of aquatic insects and oligochaetes were collected in the same way as the quantitative samples. Because the mud collector could not collect samples from the gravel bottom, we washed gravel sediment using a 60-mesh sieve or turned over the stones and directly collected the samples in the current using a screen. All samples were washed, sorted and preserved in 75% ethanol, and they were then measured (damaged specimens were only counted if the head was intact) and identified.

### 2.4. Calculation of Indicators

#### 2.4.1. Calculation of Biodiversity Indicators

To evaluate the characteristics of the species composition, species number, and species density of the ecosystem, which are important indicators of system stability, the diversity, evenness and richness values were calculated for each sample. These biodiversity indicators were computed as shown in Table 1.

#### 2.4.2. Calculation of Exergy-Based Indicators

Exergy-based indicators, including the eco-exergy (Ex) and structural eco-exergy (Exst) values, were computed as follows:

(3) $$\mathrm{Ex}=\overset{K}{\underset{k=1}{\sum }}\beta_{k}C_{k}\;$$

(4) $$\mathrm{Exst}=\overset{K}{\underset{k=1}{\sum }}\frac{\mathrm{Ex}}{C_{t}}\;$$

where Ex (kJm^−3^/kJm^−2^) is the total eco-exergy of a community, K is the total number of components selected, β_k_ is the conversion factor for component k [49,50] defined by [51], and C_k_ is the concentration of component k. Here, we used the biomass per unit of volume or area in the calculation. Exst (kJg^−1^) is the total structural eco-exergy of a community, and C_t_ is the total concentration or biomass of the organic components in the system.

#### 2.4.3. Data Analysis

To select the significant factors that affect the main community, Pearson correlation analysis was used to compare and sort water column, physical and sediment variables among the JR and BR in two seasons using SPSS version 16.0 (SPSS Inc., Chicago, IL, USA, 2007). CCA is typically used to relate the distribution of multispecies assemblages with environmental factors [52,53]. Here, CCA (Canoco 5.0, Ithaca, NY, USA) was performed to relate the environmental variables in the JR and BR to the organisation of the plankton and benthos communities. We used multidimensional scaling (MDS) to classify the biodiversity index results. MDS refers to a set of related ordination techniques that are employed for information visualisation, particularly to display the information contained in a distance matrix. Cluster analysis using the Euclidean distance was applied to classify the results of the calculated eco-exergy index, where high and low values were calculated using the average value for each community [31]. Using this method, the biodiversity and thermodynamic structures of the plankton and benthos communities were graded and analysed.

According to the grading results of biodiversity and the thermodynamic structures, we used hexagonal area maps to display the final assessment results; furthermore, the six dimensions of the hexagon represented the thermodynamic evaluation grades and community structure evaluation grade of the ecosystem (i.e., phytoplankton, zooplankton and macrobenthos, based on a 3-grade valuation method). The eco-exergy grade and community structure grade of each community was represented by the same straight line but on opposite ends of the axis of the hexagon, which represented all of the indexes of the interrelated ecological communities. Moreover, the homogeneity was high. Thus, the size of the polygon area after evaluation represented the status of each ecosystem. Based on the integrated results, larger hexagon areas represent greater system stability [40]. Furthermore, this type of system exhibits high diversity, evenness and abundance.

### 2.5. Conceptual Model

**Figure 3:**
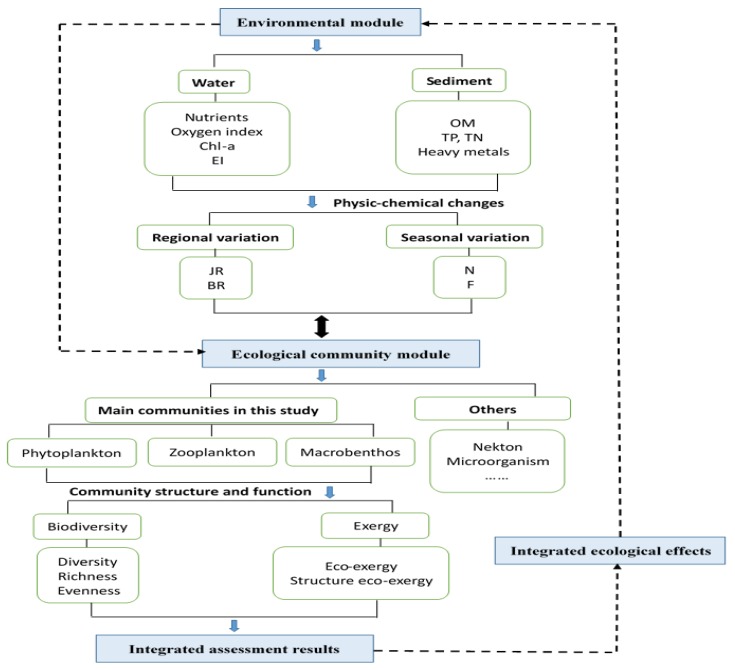
The framework of the model used to assess the relationships among the main communities and environments of urban freshwaters.

A model of the relationships among the main communities and environments of urban freshwaters was developed and applied to the JR and BR as a case study. To facilitate the visualisation and analysis, the model framework is shown in Figure 3.

## 3. Results

### 3.1. Environmental Module and Quality Standards

#### 3.1.1. Surface *Water*

The water quality (WQ) monitoring results in flood season and non-flood season are shown in Figure 2, and the integrate WQ of the JR was below that of Class V. Among all the parameters, TN and NH_3_-Nfailed to meet the standard for Class V (Supplementary Material, Table S1c). Specifically, the COD in the JR was Class I, and the CODMn was Class II~III, which indicates that the river is exposed to organic pollution. Moreover, TN was below the requirement for Class V, and NH_3_-N were below Class V (in the non-flood season) and Class IV (in the flood season). These results indicate that the discharge of high concentration nitrogen wastewater in the area seriously exceeds the WQ standard and affects the water environment of JR. Based on a comparison of the two monitoring results, the WQ of the JR in the flood season was obviously better than the WQ in the non-flood season.

In general, the WQ of the BR was Class III, which was better than the overall WQ of the JR. Specifically, the COD and BOD_5_ of the BR were Class Ⅰ, the DO was Class I–II, and the CODMn was Class II–III (Supplementary Material, Table S1d). In terms of nutrients, TP was Class II, TN was Class III, and NH_3_-N was Class I–II. Based on the calculation of the eutrophic index (EI), the BR was classified as having a mild eutrophic state (Supplementary Material, Table S2).

#### 3.1.2. Sediment

For the OM content in the sediment, analyses showed that no obvious organic pollution was found at any site except site JR2, which had displayed a high OM content. The OM ranged from 5.0 to 43.8 g/kg, and the average value was 15.14 g/kg (Table 2), which was lower than the average content in Guangzhou (26 g/kg), indicating significant horizontal differences in OM (Supplementary Material Table S3). Near the main river channel, OM is generally not deposited; thus, the OM content was low. However, the OM content was high in the river bend area where the water flow was relatively stagnant. Moreover, the spatial distribution of OM in the BR was more uniform than that in the JR. Regression analysis of the contents of OM, TP and TN in the sediment generated correlation coefficients of R^2^_(OM&TP)_ = 0.471, *p* < 0.05; R^2^_(OM&TN)_ = 0.498, *p* < 0.05; and R^2^_(TN&TP)_ = 0.941, *p* < 0.05, respectively.

The metal concentrations and results of the ecological risk assessment of the surface sediments are cited in the Supplementary Material Table S4. The contents of heavy metals in the JR and BR were much lower than those in agricultural sludge standards. Most heavy metals displayed maximum values at site JR2, with the exceptions of Hg (at BR2) and Pb (at BR1). According to the potential ecological risk assessment [54], the comprehensive pollution degree and potential ecological risk of heavy metals were greater for the JR than for the BR (Supplementary Material Table S4, Table S4a–c). The single factor evaluation of heavy metals showed that Cd contributed to the highest degree of pollution and posed a serious ecological risk (Table S4d), especially in the JR.

### 3.2. Ecological Community Module

#### 3.2.1. Community Structure and Assessment Results

**Figure 4:**
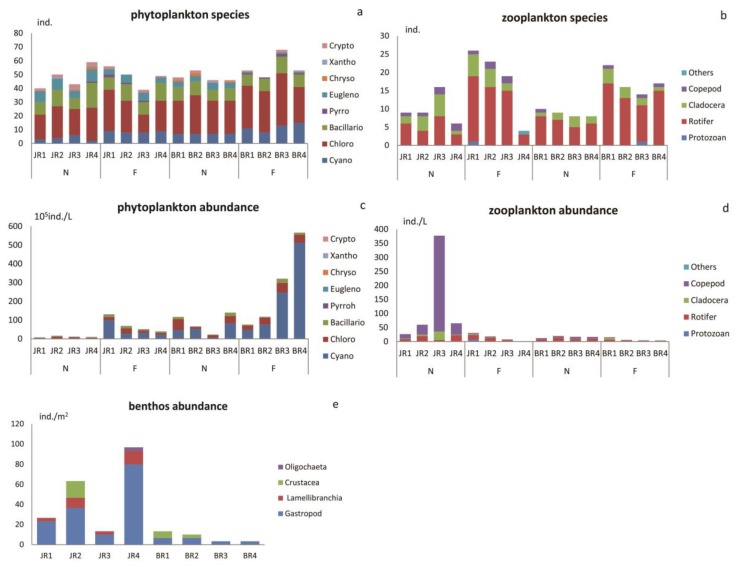
Species composition and abundance in the JR and the BR in two seasons. (**a**) phytoplankton species (ind.); (**b**) zooplankton species (ind.); (**c**) phytoplankton abundance (10^5^ ind./L); (**d**) zooplankton abundance (ind./L); and (**e**) benthos abundance (ind./m^2^).

The species composition results are shown in Figure 4 and Table 3.

The analysis of the phytoplankton species composition showed that there were 185 species of algae. Among them, 86 and 93 species were detected during the dry and wet seasons in the JR, respectively, and 79 and 102 species were detected during the dry and wet seasons in the BR, respectively. In terms of the total phytoplankton species, Chlorophyta was dominant, accounting for 40.7–57.8% of species.

A total of 66 zooplankton species were detected in the surveys, and 42 of these species were rotifers. The number of species in the rainy season was higher than that in the dry season. There were no significant differences in the number of species between the JR and the BR. Due to the aggregation of copepods in JR3, the zooplankton abundance of JR3 was the highest of all the sites. In general, the abundance of zooplankton in the dry season was larger than that in the wet season, in both the JR and the BR.

A total of 11 benthic species were identified in the surveys, and gastropods (of Mollusca) were the dominant species. The seasonal variation in benthos was not significant. The species number and abundance were higher in the JR than in the BR.

**Figure 5:**
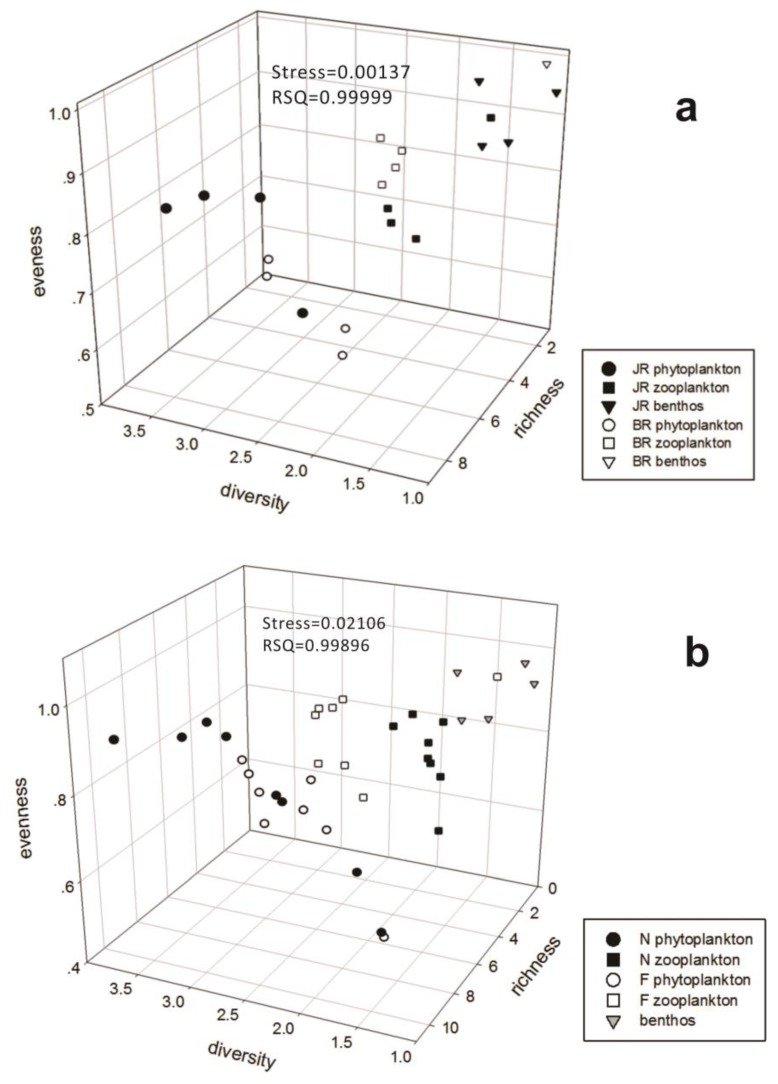
(**a**) regional and (**b**) seasonal variations in the community structure of the phytoplankton, zooplankton and benthic communities with 95% confidence limits. Stress (<0.2) and squared correlation (RSQ) (>0.6) of the final configuration were acceptable and 3D-MDS (multidimensional scaling resulting the statistical analysis) results were showed in corresponding diagrams.

We analysed each community (i.e., phytoplankton, zooplankton, and benthos) at all sites in both the non-flood season and the flood season. We established an original data matrix with all sites, using each site’s Berger-Parker index, Margalef index, Shannon index, Simpson index, and Pielou evenness index as matrix variables of MDS analysis (details in Supplementary Material Table S5). The measurement-fitting index is the stress coefficient (Stress), and, the lower the Stress, the better the degree of fitting between the graph structure and the originally provided data will be. According to Kruskal’s explanation [55], when Stress ≥ 0.2, the degree of approximation is not good (poor); Stress ≤ 0.1 is acceptable (fair); Stress ≤ 0.05 is good; Stress ≤ 0.025 is very good (excellent); and the ideal state is Stress = 0 (perfect), when the measurement of the fitted values perfectly matches the ideal value. As shown in Figure 5, we obtained the following results: (a) the primary determinant of the community structure was the community type and not the location (i.e., reservoir or river) or time (i.e., non-flood or flood season); (b) richness and diversity were displayed the trend of phytoplankton > zooplankton > benthos, and benthos exhibited higher evenness than did plankton; and (c) the regional and temporal differences in the zooplankton community structure were obvious. The regional differences were reflected by the fact that evenness in the BR was higher than that in the JR, and the temporal differences in abundance, diversity and evenness were higher during the flood season than during the non-flood season.

#### 3.2.2. Thermodynamic Structure and Assessment Results

Eco-exergy and structure exergy are two indicators that can affect various aspects of the thermodynamic structure of ecosystems. Regional and seasonal variations in each community can be obtained through formula estimation.

**Figure 6:**
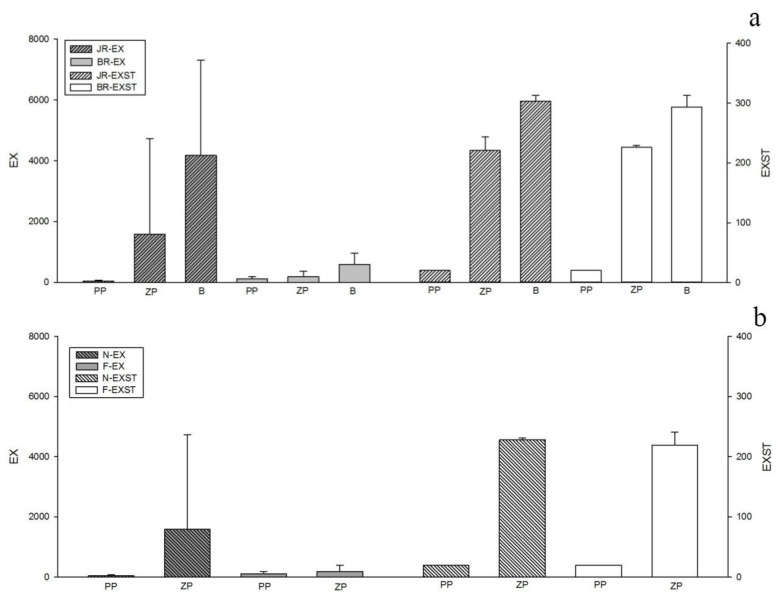
Eco-exergy indexes (**a**) in the JR and the BR (**b**) in two seasons. PP: phytoplankton, ZP: zooplankton, B: benthos.

The regional variation in the zooplankton and benthic communities differed from the variations in the phytoplankton community. Figure 6a shows that the Ex values of the zooplankton and benthic communities were higher in the JR than in the BR, but the opposite was true for the phytoplankton.

In terms of seasonal variation, Figure 6b shows that the seasonal variation in Ex and Exst were not more obvious than the regional change, especially for the benthic community. The maximum Ex value of phytoplankton appeared in the flood season, while the maximum Ex value of zooplankton appeared in the non-flood season.

## 4. Discussion

### 4.1. Relationship between the Environmental Module and the Ecological Community Module

**Figure 7:**
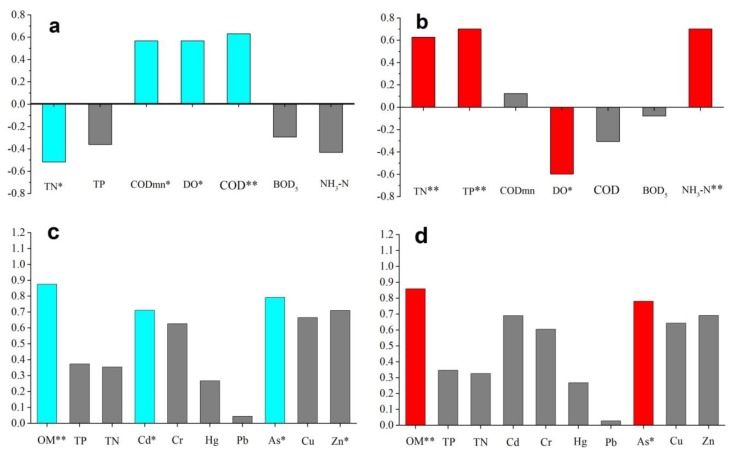
Pearson correlation analysis involving (**a**) plankton community abundance and environmental indicators; (**b**) plankton community Ex and environmental indicators; (**c**) benthic community abundance and environmental indicators; and (**d**) benthic community Ex and environmental indicators. * Significant correlation at *p* < 0.05; ** Significant correlation at *p* < 0.01; blue: the Pearson correlation between abundance and environmental indicators was significant; red: the Pearson correlation between Ex and environmental indicators was significant; grey: the Pearson correlation was not significant.

As shown in Figure 7, we selected the plankton abundance (including the phytoplankton and zooplankton communities) and water environmental indicators (Figure 7a) and the plankton exergy value and water environmental indicators (Figure 7b) at all stations in the JR and the BR in the flood and non-flood seasons for analysis. The results showed that the Pearson correlation between the abundance and Ex corresponding to WQ was obviously different. TN and DO were common significant factors; however, the plankton abundance was more easily affected by DO, the COD and other factors in water, and the Ex was affected by aquatic nutrients, such as TN, TP, NH_3_-N, as well as DO. Similarly, according to Figure 7c,d, OM was the most significant factor in the surface sediment that influenced the species abundance and exergy value of the benthic community. In addition, the benthos abundance was more easily affected by Cd, As, and Zn.

**Figure 8:**
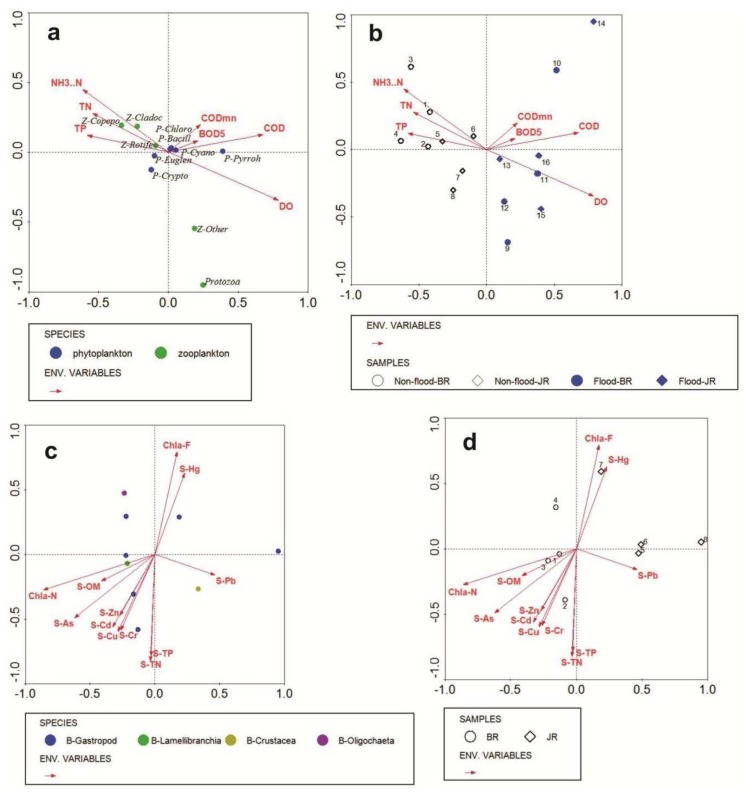
Canonical correspondence analysis involving (**a**) plankton community abundance and hydrologic environmental factors; (**b**) plankton samples and hydrologic environmental factors; (**c**) benthic community abundance and sediment environmental factors, and (**d**) benthic samples and sediment environmental factors. Environmental variables are shown by arrows, and plankton and benthos species distribution by dots. Colours represent different communities, and symbols represent different regions.

To explore the possible links between the environmental factors and the structures of the plankton and benthic communities, CCA was conducted involving the communities and environmental factors. The CCA results (Figure 8a,b) showed that there was obvious community differentiation, seasonal variation or spatial differentiation in the relationships among species and environmental factors.

Under the same water environmental conditions, phytoplankton and zooplankton exhibited obvious community differentiation (Figure 8a). The locations of the phytoplanktonic and zooplanktonic species in the CCA biplot indicated their dependence on hydrologic environmental factors. The ecological adaptation scheme was similar for species that were close together. The high plankton density and chlorophyll content in the reservoir and river (with water gates) were closely related to the nutrient contents [56], the oxygen index [57,58], and the retention time [59] of the water body. In general, as the nutrient level and primary productivity of a water body increased, the biomass and abundance of zooplankton also increased. The water retention time determined whether the zooplankton population could be maintained in the reservoir. The zooplankton abundance is generally limited when there is not sufficient time to reproduce.

Considering the “top-down effect” in freshwater ecosystems, the variations in the phytoplankton and zooplankton abundance generally differ with the season [42], and our results supported this pattern in the study area. Apart from the obvious effect of the COD on P-Pyrroh, the rest of the phytoplankton community was not affected by the WQ. Z-Copepo and Z-Cladoc were mainly concentrated in the second quadrant, which corresponded to environments with relatively high concentrations of TN and NH_3_-N. The demand for DO by Z-Other and Protozoa was higher than that by other zooplankton. In addition, the seasonal differentiation of plankton was obvious, and the flood period and drought period were limited by DO and nutrients, respectively. Moreover, compared with those in the BR, the plankton samples in the JR were mainly concentrated near the origin of coordinates that corresponded to low concentrations of various indexes of the water environment, i.e., the influence of the water environment was lower in the JR than in the BR.

The distribution patterns of macrobenthos are largely influenced by the hydrobiological and physico-chemical characteristics of the environment [60]. The diversity of functional groups of benthos provides a comprehensive reflection of environmental gradients and habitat quality [61]. Environmental parameters, such as the depth, temperature, salinity, sediment type and median grain size, were reported as important factors that influenced the macrobenthos communities in previous studies [62,63,64]. However, due to the small scope of the study area, the effects of temperature, salinity and depth were not obvious in our study; thus, we selected the environmental parameters that had significant impacts (Figure 7c,d, Pearson correlation analysis) on the benthos for further analysis. Some studies have recorded a high degree of variation in abiotic factors among inter-pool habitats; moreover, spatial and temporal variations were of greater importance than biological factors (such as food web manipulations) in terms of shaping environmental characteristics [65,66,67]. Our study indicated that physical and chemical differences occurred within the same water body and that the contents of OM and heavy metals were the main sedimentary environmental factors that affected the macrobenthos. The content of OM in sediments can indicate the degree of environmental pollution, and many sedimentary organisms feed on OM. Therefore, the content of OM is one of the important environmental factors that affects the distribution of macrobenthos. In this study, the abundance of benthos, especially Lamellibranchia, Oligochaeta, and certain gastropods, exhibited a positive correlation with total OM. In contrast, the abundance of Crustacea did not display a clear correlation with total OM (Figure 8c).

Sediments are the main repository of natural and anthropogenic metals, and the high capacity of sediments to accumulate metal compounds makes them one of the most important media to assess the contamination level in aquatic ecosystems [68]. Most contaminants leave “fingerprints” in sediments because of their stability within the sedimentary column [69]. The spatial variation patterns of heavy metal concentrations in surface sediments from the BR and the JR are presented in Figure 8c,d. The relationships between the species compositions and heavy metal contents show that the most impacted species were the gastropods. However, the heavy metal content of Pb was closely related to Crustacea. Moreover, the metal distributions differed between the JR and BR. Stations in the JR were mainly distributed in the first and fourth quadrants, and the corresponding heavy metal was Pb, which displayed a maximum concentration in the urban soil samples and likely entered the urban rivers through runoff [70]. However, the BR stations were mainly located in the second and third quadrants, and the corresponding heavy metals were Cd, As and Zn; additionally, Cd was found to be mainly associated with the acid-soluble fraction in urban soils from Guangzhou in a previous study [71]. Therefore, the regional differentiation of heavy metals was obvious. This finding is similar to those reported by Zhang et al., who stated that the concentrations of heavy metals varied among water and sediment samples and sampling stations [38,72]. OM is often viewed as a major carrier of heavy metals because of its high complexation capacity with metallic contaminants [73]. We observed that the range of abundance related to the environment was larger than that related to the eco-exergy (Pearson correlation in Figure 7, the environmental indicators related to abundance were OM, Cd, As and Zn, but only OM and As were significantly related to eco-exergy). In other words, the eco-exergy index was less sensitive to the sediment environment, and it displayed more integrity and generality than did the abundance index. Moreover, CCA showed that the OM was strongly correlated with the heavy metal contents in this study, except for those of Pb and Hg (Figure 8c), potentially because of the complex sources of OM and heavy metals in different regions [72,74].

### 4.2. Integrated Assessment Results for Spatiotemporal Changes

**Figure 9:**
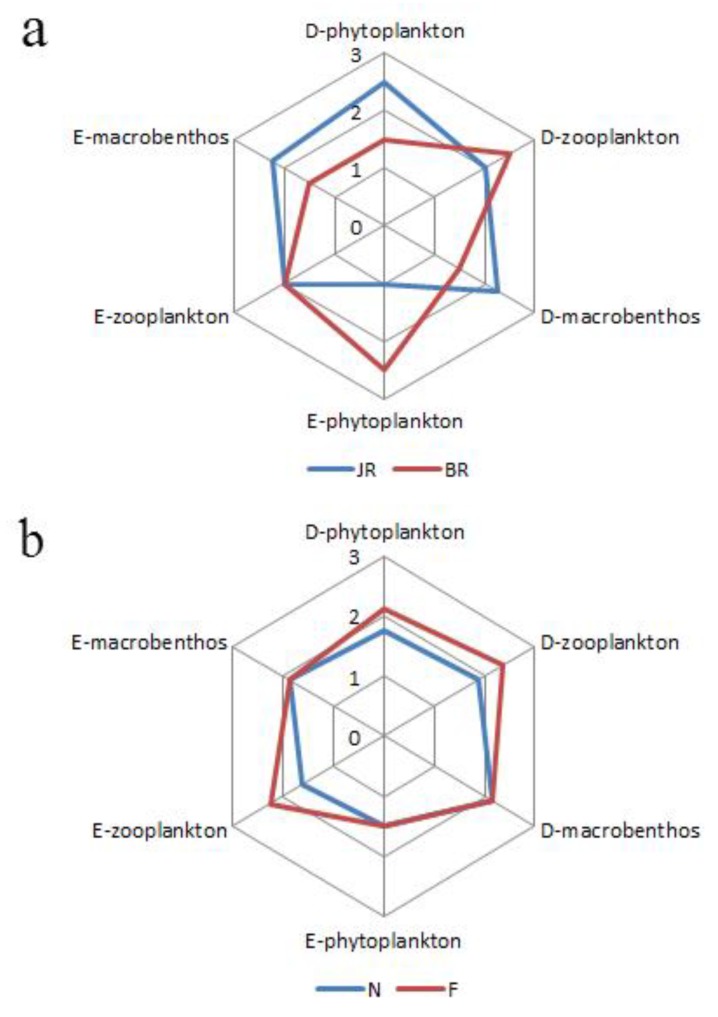
Integrative assessment results for (**a**) regional and (**b**) seasonal variations. The hexagonal area, S_i_, represents the integrative assessment results for each selected region (JR and BR) or time (N and F) for the phytoplankton, zooplankton, and macrobenthic communities.

We attempted to use a hexagonal area chart and its parameters to generate a unitary assessment of spatial and seasonal variability (Figure 9); additionally, we linked the integrative assessment status with the area size (S_i_) of the hexagon by combining community structure and thermodynamic structure. Thus, the value of S_i_ represented the status of each ecosystem, and larger values of S_i_ indicate better and more stable systems. The area calculation yielded regional values of S_JR_ ≈ 10.338 and S_BR_ ≈ 9.310, and the seasonal results were S_N_ ≈ 8.383 and S_F_ ≈ 10.834. Therefore, the structure function of the JR ecosystem was better than that of the BR ecosystem, and the flood season (F) functionality was higher than that of in the non-flood season (N).

External environmental factors can affect the integrated assessment results, which encompass the internal structure and function of the system [75,76]. Based on the assessment results reported above, the plankton eco-exergy grade was confirmed to be significantly related to the hydrologic environment in our study. Under satisfactory hydrologic environment conditions, the eco-exergy grade was relatively high, e.g., WQ was better in the BR than in the JR, and WQ was better in the F period than in the N period; thus, the eco-exergy grade of the BR was greater than that of the JR, and the grade in the F period was higher than that in the N period. However, the community structure grade of phytoplankton, which encompassed the diversity, evenness and abundance indexes, was not always positively correlated with the eco-exergy indicators. It displayed good consistency in high eco-exergy grade systems, such as the zooplankton and macrobenthic communities in the investigated ecosystem. This result indicated that eco-exergy and biodiversity differed greatly in the low-grade systems of the eco-exergy grade assessment, likely because the higher eco-exergy grade community reached a threshold diversity level for advanced organisms, and the system was mature [77]. The structure and function of the system can be maintained at a high grade for a long time when the system reaches this abundance threshold. Therefore, this type of system is in a relatively balanced state [78].

For benthos, the higher eco-exergy values found in the JR can be explained by the higher concentrations of available nutrient inputs, which resulted in the increased use of available resources to build a more complex dissipative structure [79]. This finding corresponded to growth in the network and to OM enrichment and biomass storage [80]. Although N pollution in the JR exceeded the standard in WQ assessment, the TN concentration in sediments of the JR were just slightly higher than that of the BR owning to the differences of detention time, enrichment conditions and flow velocity of the JR and BR. Moreover, there is more energy available to benthic assemblages in a system where non-excessive nutrient enrichment occurs, and this scenario leads to an increase in eco-exergy [81]. The benthos have a relatively fixed living space and a relatively long life cycle; thus, the response of benthos to seasonal change was not obvious in the present study.

In brief, we obtained two major findings from the integrated assessment results. First, the structure and functional status of the high eco-exergy grade community in the ecosystem had a considerable influence on the regional changes in the integrated assessment results. In terms of regional variations (Figure 9a), S_JR_ was slightly greater than S_BR_ because the D-phytoplankton, E-macrobenthos and D-macrobenthos in the JR were higher than those in the BR. Additionally, the benthic eco-exergy grade and biodiversity grade contributed to these variations. Researchers have shown that the reliability of the eco-exergy grade is generally dependent on the availability of detailed β values, which can be computed from information stored in genes [82,83]. Compared to the plankton communities that exhibited low β values and the eco-exergy grades based on the number and density of species in the community, the benthos eco-exergy grade was determined by the proportions of high-β molluscs and crustaceans [40]. In addition, our study confirmed that benthic biodiversity and eco-exergy were positively correlated with the OM content (g/kg) in the sedimentary environment. Notably, the OM content in the JR (22.53 ± 19.73) was higher than that in the BR (8.01 ± 2.50), which indicated that sufficient OM in sediment can support a more balanced macrobenthic system and have an effect on the integrated assessment results, although OM may also be associated with the risk of heavy metal pollution.

Second, the significant seasonal variations in the plankton communities affected the seasonal variations in the holistic assessment; therefore, the overall plankton community is an effective indicator of seasonal ecosystem changes. Moreover, S_F_ was obviously greater than S_N_ (Figure 9b). The grades of the D-phytoplankton, D-zooplankton and E-zooplankton in the F period were higher than those in the N period. The F periods associated with increases in the water volume, runoff, turbidity, and nutrient levels, which are required for the normal growth and development of phytoplankton [84]. Moreover, phytoplankton species are able to cross most local-scale dispersal barriers at higher migratory rates during the F period [85]; therefore, phytoplankton are better dispersed during this period, and D-phytoplankton are more abundant than during the N period. D-zooplankton was accompanied by the growth of D-phytoplankton, although the zooplankton abundance was not always large in this period [86]. Moreover, the structure eco-exergy of zooplankton was generally higher than that of phytoplankton; thus, the E-zooplankton was higher during the F period. These results imply that the structure and function of the plankton communities in urban rivers and reservoirs are significantly different in seasons, which affects the seasonal variations in the integrated assessment results.

Generally, the system structure and function of the JR and F were more stable than those of the BR and N, and the energy gained and stored was greater. Therefore, if the system was adjusted by changing the environmental conditions, the former needs more energy and power; as a result, the regulation and control of the ecosystem are relatively difficult to achieve in the JR and the F period. This study may provide theoretical support for the management and restoration of freshwater environments and ecosystems in the region.

## 5. Conclusions

An analysis of the variation in the integrated assessment results was conducted among environmental factors in the JR and the BR. The findings revealed that external environmental factors can affect the integrated assessment results, as mainly reflected by the internal structure and function of the system. The plankton community composition was largely restricted by hydrologic environmental variables, particularly the nutrient concentrations and oxygen index, and the development of the macrobenthic community was mainly affected by OM and heavy metals. In terms of regional variations, S_JR_ was slightly greater than S_BR_ based on the macrobenthic eco-exergy grade and biodiversity grade. However, the significant seasonal variations in the plankton community affected the seasonal variations in the integrated assessment results; thus, S_F_ was obviously greater than S_N_. Overall, the current study describes a basic framework that can be used to establish links among environmental factors and ecosystems and to conduct integrated assessments of urban river and reservoir ecosystems.

## Figures and Tables

**Table 1:** Calculation method for the biodiversity index [45,46,47,48].

Biodiversity	Indexes	Calculation Formulas	Comments
Abundance	Species abundance	S	S = Number of species in the community
Diversity	Shannon Wiener Index (base e)	$H_{e}=-\overset{S}{\underset{i=1}{\sum }}P_{i}\times \ln P_{i},\;P_{i}$ = $\frac{n_{i}}{N}$	N = Number of individuals in the community
	Simpson Index	$D_{1-D}=1-\overset{S}{\underset{i=1}{\sum }}P_{i}^{2}$, $P_{i}^{2}=\frac{n_{i}\left(n_{i}\right)}{N\left(N-1\right)}$	
Richness	Margalef Index	$d_{\mathrm{Ma}}=\frac{\left(S-1\right)}{\ln N}$	
Evenness	Pielou’s Index	J = H_/lnS	

**Table 2:** The investigation results for OM (organic matter), TP (total phosphorus) and TN (total nitrogen) content in the sediment.

Investigation Station and Value	JR1	JR2	JR3	JR4	BR1	BR2	BR3	BR4	Average Content
OM (g/kg)	5.00	43.80	6.50	33.80	10.20	10.10	6.20	5.50	15.1
TP (g/kg)	0.36	0.91	0.30	0.38	0.42	0.62	0.28	0.29	0.44
TN (g/kg)	0.34	1.05	0.25	0.31	0.41	0.53	0.23	0.35	0.43

**Table 3:** Statistical characteristics of community components.

Species	Species Number	Abundance				Dominant Species	
		Region	Mean	Non-Flood	Flood	Non-Flood	Flood
Phytoplankton	185	7.53–566.70 × 10^5^ ind./L	129.82 × 10^5^ ind./L	Low	High	chlorophyta	cyanobacteria
Zooplankton	66	0.90–377.60 ind./L	42.64 ind./L	High	low	Rotifer	Rotifer
Benthos	11	3.33–96.67 ind./m^2^	33.00 ind./m^2^	--		gastropods	

## Supplementary Materials

The following are available online at http://www.mdpi.com/1660-4601/15/10/2302/s1. S1: Water environmental quality standards and Five classes of surface water bodies (I–V), S2: The eutrophication evaluation of lakes, S3: The background value of OM content in different sediment types in Guangzhou City and the classification of OM pollution degree, S4: The heavy metal concentrations and ecological risk assessment results, S5: The composition of original data matrix of MDS analysis.
