# Supplementary material for: Relationship between the Main Communities and Environments of an Urban River and Reservoir: Considering Integrated Structural and Functional Assessments of Ecosystems
Source: Int J Environ Res Public Health. 2018 Oct 19;15(10):2302. doi: 10.3390/ijerph15102302 (PMC6210642; doi:10.3390/ijerph15102302)
Supplement: Supplementary file 1 [file ijerph-15-02302-s001.pdf]

Supplementary material

S1: Water environmental quality standards and Five classes of surface water bodies (Ⅰ-Ⅴ)

According to the demand of surface water quality of national environmental policy, The first revision was made in 1988 to form the version of GB3838-88. It was then converted to GHZB 1-1999 in 1999 after 11 years of its implementation. In 2002, the standards experienced the third revision and became the latest version (GB3838-2002) being effective until now. After these 3 revisions, National Surface Water Quality Standards developed a comprehensive system in which nutrient indicator values in lakes and reservoirs have been identified. GB 3838-2002 has become the core for national water environmental monitoring, and plays a very important role in water pollution prevention regulation and water environmental management system.

Table S1-a Basic information of Water environmental quality standards (GB3838-2002) (SEPA, 2002).

Surface WQS	Standard	GB3838-2002
	Classfication	5 Classes
Indicators	**Basic parameters**	**24**
	**Organic chemical standards**	**80**

	Lake eutrophication	5
Assessment method		Multi-factors comprehensive evaluation

Table S1-b Five classes of surface water bodies (Ⅰ-Ⅴ).

**Indicators**					**Classes**		
**unit: mg/L**			Ⅰ	Ⅱ	Ⅲ	Ⅳ	Ⅴ
**TN**		⩽	0.2	0.5	1.0	1.5	2.0
**TP**	**River**	⩽	0.02	0.1	0.2	0.3	0.4
	**Lake and reservoirs**	⩽	0.01	0.025	0.05	0.1	0.2
**NH_3-N**		⩽	0.15	0.5	1.0	1.5	2.0
**DO**		⩾	90% or 7.5	6	5	3	2
**COD**		⩽	15	15	20	30	40
**BOD_5**		⩽	3	3	4	6	10
**COD_{Mn}**		⩽	2	4	6	10	15

Parameters values were determined in accordance with the water environmental protection objective of aquatic life and human health for both water body and the recharge sources (Su et al., 2017). This standard effectively acted as preventing water pollution, ensuring human health, protecting resource, maintaining ecological balance, and conserving sustainable economic development. It could be applied to rivers, lakes, reservoirs, and other water basins, and became a major regulation for environmental planning, environmental management, water quality assessment, and pollutant discharge (SEPA, 2002).

Table S1-c Monitoring results of the water quality indexes and Five classes of the JR.

		JR1		JR2		JR3		JR4	
		N	F	N	F	N	F	N	F
**TN**	**value**	3.31	1.70	3.78	2.06	4.58	2.52	3.44	2.48
	**class**	-Ⅴ	Ⅴ	-Ⅴ	-Ⅴ	-Ⅴ	-Ⅴ	-Ⅴ	-Ⅴ
**TP**	**value**	0.10	0.06	0.13	0.07	0.16	0.08	0.11	0.08
	**class**	Ⅲ	Ⅱ	Ⅲ	Ⅱ	Ⅲ	Ⅱ	Ⅲ	Ⅱ
**CODmn**	**value**	3.90	3.40	4.30	4.00	3.90	3.50	3.10	2.30
	**class**	Ⅱ	Ⅱ	Ⅲ	Ⅱ	Ⅱ	Ⅱ	Ⅱ	Ⅱ
**DO**	**value**	4.90	7.20	4.70	6.50	4.20	6.60	4.70	6.80
	**class**	Ⅳ	Ⅱ	Ⅳ	Ⅱ	Ⅳ	Ⅱ	Ⅳ	Ⅱ
**COD**	**value**	10.00	10.00	10.00	12.50	10.00	10.00	10.00	13.70
	**class**	Ⅰ	Ⅰ	Ⅰ	Ⅰ	Ⅰ	Ⅰ	Ⅰ	Ⅰ
**BOD5**	**value**	3.00	3.40	3.00	5.30	2.40	5.40	3.00	2.20
	**class**	Ⅱ	Ⅲ	Ⅱ	Ⅳ	Ⅱ	Ⅳ	Ⅱ	Ⅱ
**NH3—N**	**value**	2.01	0.30	2.29	1.03	2.97	0.93	1.74	0.06
	**class**	-Ⅴ	Ⅱ	-Ⅴ	Ⅳ	-Ⅴ	Ⅲ	Ⅴ	Ⅰ

-Ⅴ: below Ⅴ

Table S1-d Monitoring results of the water quality indexes and Five classes of the BR.

		BR1		BR2		BR3		BR4	
		N	F	N	F	N	F	N	F
**TN**	**value**	0.87	0.81	0.82	0.95	0.77	0.96	0.93	0.69
	**class**	Ⅲ	Ⅲ	Ⅲ	Ⅲ	Ⅲ	Ⅲ	Ⅲ	Ⅲ
**TP**	**value**	0.04	0.05	0.04	0.04	0.03	0.05	0.04	0.05
	**class**	Ⅲ	Ⅲ	Ⅲ	Ⅲ	Ⅲ	Ⅲ	Ⅲ	Ⅲ
**CODmn**	**value**	3.40	3.40	3.40	3.70	3.50	4.50	3.40	4.70
	**class**	Ⅱ	Ⅱ	Ⅱ	Ⅱ	Ⅱ	Ⅲ	Ⅱ	Ⅲ
**DO**	**value**	6.50	7.60	7.00	7.80	6.90	7.80	6.90	7.70
	**class**	Ⅱ	Ⅰ	Ⅱ	Ⅰ	Ⅱ	Ⅰ	Ⅱ	Ⅰ
**COD**	**value**	11.50	12.10	11.10	13.60	10.40	12.80	10.00	14.60
	**class**	Ⅰ	Ⅰ	Ⅰ	Ⅰ	Ⅰ	Ⅰ	Ⅰ	Ⅰ
**BOD5**	**value**	2.20	2.00	2.10	2.00	2.50	2.00	2.00	2.00
	**class**	Ⅰ	Ⅰ	Ⅰ	Ⅰ	Ⅰ	Ⅰ	Ⅰ	Ⅰ
**NH3—N**	**value**	0.33	0.13	0.28	0.17	0.24	0.17	0.26	0.25
	**class**	Ⅱ	Ⅰ	Ⅱ	Ⅱ	Ⅱ	Ⅱ	Ⅱ	Ⅱ

S2:The eutrophication evaluation of lakes

The eutrophication evaluation of lakes is evaluated by Technological regulations for surface water resources quality assessment (SL395-2007). Nutritional status evaluation items include total phosphorus (TP), total nitrogen (TN), chlorophyll a (chla), permanganate index (PI) and transparency (Tr). Nutritional status evaluation items include total phosphorus, total nitrogen, chlorophyll a, permanganate index and transparency. The formula for evaluating the nutritional status of lakes and reservoirs is as follows:

$$\mathrm{EI} = \sum_{n=1}^{N} E_n \,/\mathrm{N}$$

Where, EI is eutrophic index; En is the assignment of nutritional status evaluation items; N is the number of evaluation items.

Table S2-a Evaluation standard and classification of Lake nutrition state

**EI**		**En**	**TP (mg/L)**	**TN (mg/L)**	**Chla (mg/L)**	**PI (mg/L)**	**Tr (m)**
**Oligotrophic condition**		10	0.001	0.020	0.0005	0.15	10
**0＜EI≤20**		20	0.004	0.050	0.0010	0.4	5.0
**Mesotrophic condition**		30	0.010	0.100	0.0020	1.0	3.0
**20＜EI≤50**		40	0.025	0.300	0.0040	2.0	1.5
		50	0.050	0.500	0.010	4.0	1.0
	**Light 50＜EI≤60**	60	0.10	1.0	0.026	8.0	0.5
**Eutrophic**	**Middle 60＜EI≤80**	70	0.20	2.0	0.064	10	0.4
**condition**		80	0.60	6.0	0.16	25	0.3
	**Severe 80＜EI≤100**	90	0.90	9.0	0.40	40	0.2
		100	1.30	16.0	1.0	60	0.12

Table S2-b Monitoring results of the water quality indexes and EI of the BR

Sites	BR1		BR2		BR3		BR4	
**Season**	N	F	N	F	N	F	N	F
**TN**	0.87	0.81	0.82	0.95	0.77	0.96	0.93	0.69
**TP**	0.04	0.05	0.04	0.04	0.03	0.05	0.04	0.05
**PI**	3.4	3.4	3.4	3.7	3.5	4.5	3.4	4.7
**Chla**	0.035	0.018	0.042	0.022	0.043	0.035	0.039	0.041
**EI**	54	53	54	54	53	56	54	55

S3: The background value of OM content in different sediment types in Guangzhou City (Wen et al., 1996; Li et al., 2005) and the classification of OM pollution degree.

Table S3-a: The background value of OM content in different sediment types in Guangzhou City

Index and background value		River	Soil	Sewage sludge	Vegetable soil	Paddy soil
The average content in Guangzhou	OM	51.5	26	128.4	50.8	28.81
	OC	29.89	15.29	75.53	29.88	16.94

Table S3-b: The classification of OM pollution degree.

Classification of OM pollution degree	OM	0-34	34-51	51-68	>68
	Pollution Degree	Unpolluted	Light pollution	Medium pollution	Heavy pollution

S4:The heavy metal concentrations and ecological risk assessment results

Table S4-a: The heavy metal concentrations results in surface sediments.

Sampling sites	Cd (mg/kg)	Cr (mg/kg)	Hg (mg/kg)	Pb (mg/kg)	As (mg/kg)	Cu (mg/kg)	Reference
**JR1**	0.34	31.15	0.09	55.49	10.08	13.41	↑
**JR2**	2.27	93.35	0.09	79.67	20.81	97.56	
**JR3**	0.34	24.72	0.04	41.26	6.50	9.76	
**JR4**	1.08	49.98	0.16	69.72	12.68	41.46	
**BR1**	0.36	29.73	0.11	211.99	2.60	15.85	Our study
**BR2**	0.47	37.91	0.18	72.56	0.98	15.85	
**BR3**	0.07	34.69	0.09	12.80	0.65	2.44	
**BR4**	0.45	34.33	0.11	45.53	2.93	24.39	
**Max**	2.27	93.35	0.18	211.99	20.81	97.56	
**Min**	0.07	24.72	0.04	12.80	0.65	2.44	↓
**Average**	0.67	41.98	0.11	73.63	7.15	27.59	
**Soil background value in Guangzhou**	0.144	60.35	0.161	47.08	—	21.81	Wen et al., 1996
**Agricultural sludge standard**	5	600	5	300	75	250	GB 4284-84
**Pearl River, China**		86.09		43.28		46.08	Zhou et al., 2004
**Pearl River, China**				39.05		40.09	Li et al., 2000

Table S4-b: The heavy metal comprehensive polluted degree

Sampling sites	C_{Cd}	C_{Cr}	C_{Hg}	C_{Pb}	C_{As}	C_{Cu}	C_{Zn}	C_d
**JR1**	8.427	0.617	1.119	1.541	1.133	0.789	1.626	15.252
**JR2**	56.742	1.849	1.119	2.213	2.339	5.739	3.826	**73.826**
**JR3**	8.427	0.490	0.560	1.146	0.731	0.574	1.339	13.266
**JR4**	26.966	0.990	1.959	1.937	1.425	2.439	2.726	**38.441**
**BR1**	8.989	0.589	1.399	5.889	0.292	0.933	1.674	19.764
**BR2**	11.798	0.751	2.239	2.016	0.110	0.933	2.248	20.093

**BR3**	1.685	0.687	1.119	0.356	0.073	0.143	0.430	4.494
**BR4**	11.236	0.680	1.399	1.265	0.329	1.435	1.817	18.161
**Average**	16.784	0.831	1.364	2.045	0.804	1.623	1.961	25.412

Table S4-c: The heavy metal potential ecological risk.

**Sampling sites**	E_{Cd}	E_{Cr}	E_{Hg}	E_{Pb}	E_{As}	E_{Cu}	E_{Zn}	**RI**
**JR1**	252.809	1.234	44.776	7.707	11.327	3.945	1.626	323.424
**JR2**	1702.245	3.697	44.776	11.066	23.385	28.694	3.826	**1817.690**
**JR3**	252.809	0.979	22.388	5.731	7.308	2.869	1.339	293.423
**JR4**	808.988	1.979	78.358	9.683	14.250	12.195	2.726	**928.179**
**BR1**	269.663	1.178	55.970	29.443	2.923	4.663	1.674	365.514
**BR2**	353.933	1.501	89.552	10.078	1.096	4.663	2.248	463.070
**BR3**	50.562	1.374	44.776	1.778	0.731	0.717	0.430	100.369
**BR4**	337.079	1.360	55.970	6.323	3.289	7.174	1.817	413.011
**Average**	503.511	1.663	54.571	10.226	8.039	8.115	1.961	588.085

Table S4-d: The standards of ecological risk assessment.

C^i_j	**Single factor index**	C_d	**Comprehensive polluted degree**	E^i_j	**Potential ecological risk of single factor**	**RI**	**Comprehensive potential ecological risk**
<1	Low	<5	Low	<40	Light	<150	Light
1-3	Medium	5-10	Medium	40-80	Medium	150-300	Medium
3-6	High	10-20	High	80-160	Heavier	300-600	Heavy
≥6	Very high	≥20	Very high	160-320	Heavy	≥600	Serious
				≥320	Serious		

S5: The composition of original data matrix of MDS analysis

Table S5-a: Formulae for matrix variables of MDS.

Indices	Formulae	Explanation	References
**Berger-Parker index (d)**	$\mathbf{d = 1/\frac{n_{max}}{N}}$	n_{max}: the number of the most species	Berger and Parker, 1970
**Margalef index (d_{Ma})**	$\mathbf{d_{Ma} = \frac{S-1}{lnN}}$		Margalef, 1958
**Simpson index (λ)**	$\boldsymbol{\lambda = \sum_{i=1}^{S} P_i^2}$	$\mathbf{P_i^2 = \frac{n_i(n_i-1)}{N(N-1)}}$	Simpson, 1949
**Simpson index (D)**	$\mathbf{D = 1 - \sum_{i=1}^{S} P_i^2}$	$\mathbf{P_i^2 = \frac{n_i(n_i-1)}{N(N-1)}}$	Greenberg, 1956
**Simpson index (D_r)**	$\mathbf{D_r = 1/\sum_{i=1}^{S} P_i^2}$	$\mathbf{P_i^2 = \frac{n_i(n_i-1)}{N(N-1)}}$	Hill, 1973
**Shannon index (H_e')**	$H_e' = -\sum_{i=1}^{S} P_i \times \ln P_i$	$\mathbf{P_i = \frac{n_i}{N}}$	Shannon, 1948
**Shannon index (H_2')**	$H_2' = -\sum_{i=1}^{S} P_i \times \log P_i$	$\mathbf{P_i = \frac{n_i}{N}}$	Shannon, 1948
**Pielou's Index (J_e)**	$J = H_e' / \ln S$		Pielou, 1966

S: species number; N: sum of the individual number of all species; n_i: number of species i

Table S5-b: Take regional variation matrix of MDS analysis as an example.

Communities	Indices	JR1	JR2	JR3	JR4	BR1	BR2	BR3	BR4
phytoplankton	d	4.416	8.769	5.815	10.889	4.500	2.988	4.255	2.636
	d_{Ma}	6.791	7.235	5.973	8.421	6.518	6.192	6.727	5.519
	λ	0.248	0.051	0.078	0.054	0.097	0.246	0.100	0.209
	D	0.752	0.949	0.922	0.946	0.903	0.754	0.900	0.791
	D_r	10.662	20.562	15.664	25.086	10.422	5.280	10.123	5.280

	H_e'	2.491	3.292	3.027	3.385	2.855	2.228	2.832	2.317
	H_2'	3.593	4.749	4.367	4.883	4.118	3.215	4.085	3.343
	J_e	0.655	0.841	0.814	0.848	0.728	0.570	0.705	0.594
zooplankton	d	3.173	3.205	3.846	2.669	3.750	4.612	4.720	4.854
	d_{Ma}	3.665	3.357	3.616	1.765	3.671	3.276	3.060	3.450
	λ	0.170	0.193	0.156	0.151	0.133	0.131	0.126	0.107
	D	0.830	0.807	0.844	0.849	0.867	0.869	0.874	0.893
	D_r	6.682	5.391	8.451	7.485	7.521	8.912	9.329	10.491
	H_e'	2.234	2.009	2.207	1.578	2.291	2.229	2.206	2.347
	H_2'	3.223	2.898	3.185	2.276	3.306	3.216	3.183	3.386
	J_e	0.761	0.705	0.735	0.895	0.802	0.824	0.849	0.876
benthos	d	2.000	3.800	2.000	2.417	2.000	3.000	1.000	1.000
	d_{Ma}	1.924	1.698	1.443	1.782	1.443	1.820	(-)	(-)
	λ	0.214	0.146	0.167	0.217	0.167	0.000	(-)	(-)
	D	0.786	0.854	0.833	0.783	0.833	1.000	(-)	(-)
	D_r	4.667	6.840	6.000	4.614	6.000	(-)	(-)	(-)
	H_e'	1.386	1.709	1.040	1.643	1.040	1.099	0.000	0.000
	H_2'	2.000	2.465	1.500	2.370	1.500	1.585	0.000	0.000
	J_e	0.861	0.954	0.946	0.844	0.946	1.000	(-)	(-)

The index value is the average of two quarters (N and F).

References

Berger, W.H., Parker, F.L.,1970. Diversity of planktonic foraminifera in deep-sea sediments.Science.1345-1347.

Control standards for pollutants in sludges from agricultural use, GB4284-84, 1985 (In Chinese).

Greenberg, J.H., 1956. The measurement of linguistic diversity.Language. 32: 105-119.

Hill, M.O.,1973.Diversity and evenness unifying notation and its consequences.Ecology.54, 427-431.

Li, X., Wai, O.W.H., Li, Y.S., Coles, B.J., Ramsey, M.H., Thornton, I., 2000. Heavy metal distribution in sediment profiles of the Pearl River estuary, south china. Appl. Geochem. 15, 567-581.

Margalef, R., 1958. Information theory in ecology. General Systematics. 3, 36-71.

Pielou, E.C., 1966. The measurement of diversity in different types of biological collections. J.Theor.Biol. 13, 131-144.

SEPA, 2002. Environmental Quality Standard for Surface Water (GB3838-2002). State Environmental Protection Administration of China, Beijing of Regula.

Shannon, C.E., 1948. A mathematical theory of communication.Bell System Technical Journal, 27, 379-423.

Simpson, E.H., 1949. Measurement of biodiversity.Nature.163, 688.

Su, J., Ji, D., Lin, M., Chen, Y., Sun, Y., Hou, S., Zhu, J., Xi, B., 2017. Developing surface water quality standards in China. Resour. Conserv. Recy. 117, 294-303.

Wen, Y.M., Wei, Z.T., 1996. The Concentration and Availability of Heavy Metals inMunicipal Sewage Sludge and Soil in Guangzhou. Acta Scientiarum Naturalium Universitatis Sunyatseni, 35, 217–221 (In Chinese with English abstract).

Zhou, H. Y., Peng, X. T., Pan, J. M., 2004. Distribution, source and enrichment of some chemical elements in sediments of the Pearl River estuary, china. Cont.Shelf Res. 24, 1857-1875.
